# Papillary and Callous Scales in the Integument of Agamid Lizards (Agamidae, Sauria) as a Phenomenon of Extraordinary Development of the Corneous Layers

**DOI:** 10.3390/ani15050743

**Published:** 2025-03-05

**Authors:** Tatjana N. Dujsebayeva, Natalia B. Ananjeva, Nasrullah Rastegar-Pouyani, Awadh M. Al-Johany, Daniel A. Melnikov

**Affiliations:** 1 Department of Ornithology and Herpetology, Institute of Zoology, Almaty 050060, Kazakhstan; tatjana.dujsebayeva@zool.kz; 2 Department of Herpetology Zoological Institute, 199034 Saint-Petersburg, Russia; persicus@zin.ru; 3 Department of Zoolgy, Faculty of Sciences, Razi University, Kermanshah 6714967346, Iran; 4 Department of Zoology, College of Sciences, King Saud University, Riyadh P.O. Box 2455, Saudi Arabia; ajohany@ksu.edu.sa

**Keywords:** Squamata, *Acanthocercus*, *Laudakia*, epidermal glands, dermal papillae, LM, SEM

## Abstract

Scaled integument of six species of the genus *Acanthocercus* and *Laudakia nupta*, family Agamidae was studied using light and scanning electron microscopy. Gross observation revealed the presence of two types of modified scales in the males. The enlarged scales covered with an extremely thick *β*-corneous layer were detected in the dorsal and ventral surfaces of the tail base and on the palmar and plantar limb surfaces of all species. After detachment of the *β*-layer, the surface of such scales was covered with high papillae (“papillary scales”). The callous scales were found in the precloacal region of *Acanthocercus* species and in both precloacal and mid-ventral regions of *L. nupta*. Modified scales were found in some females and subadut specimens, and absent in juveniles. A prominent papillary layer characterized the dermis of both scale types. It was assumed that well-developed dermal papillae in such scales expended the total surface area of stratum germinativum and created a pool of proliferated cells in the interpapillary loops to increase the production of differentiating keratinocytes. These processes were undoubtedly associated with the formation of a thick and resistant corneous layer that distinguished both types of scales. Functional role, a taxonomic value, and analogies with normal and pathological epidermis in birds and mammals are discussed.

## Simple Summary

Using visual inspection, light and scanning electron microscopy, we studied the integument of six species of the genus *Acanthocercus* and *Laudakia nupta* (family Agamidae). Two types of unusual scales were found. Scales covered with an extremely thick corneous layer were detected around the tail base and on the palmar and plantar limb surfaces of all species. When the corneous layer was removed, the surface of such scales remained covered with numerous high papillae (“papillary scales”). Non-overlapping callous-like scales (“callous scales”) were placed in front of cloaca of all *Acanthocercus* species and in front of the cloaca and in the central part of the belly of *L. nupta*. Modified scales were certainly male characteristics, developed to a lesser degree and only in some females and subadut specimens, and absent in juveniles. The dermis of modified scales had a well-developed papillary layer that expended the total surface area of stratum germinativum, created a pool of proliferated cells, and promoted a higher production of intensively differentiating keratinocytes with final formation of thick corneous layer that distinguish both types of scales. The functions of modified scales in lizards and some analogies with specialized areas of normal and pathological epidermis of birds and mammals are discussed.

## 1. Introduction

The qualitative and quantitative characteristics of scalation are of great importance in the taxonomy of squamate reptiles, including the species of the family Agamidae. Among the most important scalation characteristics of the lizards of the genera *Acanthocercus* and *Laudakia* are large-keeled caudal scales forming more or less distinct annuli and the presence of modified glandular scales in the ventral integument of the body [1,2].

The functional significance of keeled caudal scales with distinct annuli is still a subject of debate. The prevailing view suggests the anchoring role of a powerful spiny tail in lizards fleeing from predators in rocky crevices [3,4]. In the key to the species of the genus *Agama* for *Agama atricollis* (*=Acanthocercus atricollis*) [1], Boulenger initially noted a special characteristic of the large and rigid caudal scales of these lizards: “tail scales are strongly keeled, and forming rather regular annuli, those on the upper surface much enlarged, with denticulated edge”. The identified scales “with jagged margins” were recorded in some species of agamid lizards: *Laudakia melanura*, *L. nupta*, *L. sacra*, *Paralaudakia erythrogaster*, *Acanthocersus annectans* [5,6]. The scales “with papillary structure and jagged margins” were described” in *Paralaudakia caucasia* and *P. erythrogastra* but not in *P. himalayana*, *P. lehmanni* and *P. stoliczkana* [7]. A comparative study of the scale integument in ten species of the former genus *Laudakia* and three species of the genus *Acanthocercus* revealed significant heterogeneity in the degree of papillary scale development in *Laudakia*, including their complete absence in some species, but presence in all examined species of *Acanthocercus* (*A. atricollis*, *A. annectans*, and *A. cyanogaster*) [8]. In the same work, a sexual dimorphism in the manifestation of papillary scales in all the species studied and a geographic variation in their development in *P. caucasia* populations have been noted. We do not know any other works besides those listed where the mentioned scales would be described. There is an absence of work on the fine morphology of these structures.

The modified glandular scales—the “callose preanal scales” were at first used as a diagnostic characteristic of the former collective genus *Agama* [1] and clearly distinguished them from the femoral and preanal follicular glands (“pores”). Tölg [9] together with the detailed histological description of these structures in *Agama inermis* (=*Trapelus mutabilis*) introduced some confusion in terminology by naming them “Präanale Papillarorgane”. Afterwards [10] this term was repeatedly used in its French translation—“Organes papillaires”. All other researchers described these structures synonymous with Boulenger’s term: “callose” or “callous” scales [11,12,13,14,15], “callous pads” [16] or “callous glands” [17].

Such structures were classified based on their morphology [17] as “generation glands”, in which the secretory product is laid down, matures and is then released to the environment in synchrony with the normal epidermal turnover [18,19]. Since callous scales were normally more developed in males than in females, the researchers have suggested their possible chemocommunicative role in male behavior as well as their use in self-, sex and species recognition and orientation [14,15,20,21,22,23]. The fine structure of callous scales has been studied in only a few species of agamids: *Agama inermis* (=*Trapelus mutabilis*) [9,10], *Agama agama* [16], *Paralaudakia* (former *Laudakia*) *caucasia* and *P. himalayana* [14,15].

This work is the third in our series of morphological studies of the integumentary derivatives of lizards [15,24]. Here, we have significantly expanded the list of agamid species studied to identify modified scales in their integument including some species which were briefly mentioned earlier [25]. For the first time we have described the morphology of papillary scales using light microscopy (LM) and scanning electron microscopy (SEM), and provided data on the histology of callous scales in *Laudakia nupta*, which has not been previously described in this regard. We succinctly analyzed the presence of modified scales depending on the sex and age of the lizards and expressed additional hypotheses regarding the functional significance of these structures.

## 2. Materials and Methods

### 2.1. Sample Collection

We examined the integument of eight species of lizards of the genera *Acanthocercus* and *Laudakia nupta*, including the belly, cloacal region, and palmar and plantar surfaces of the limbs. The specimens were obtained from the Museum Alexander Koenig, Bonn, Germany (ZFMK, N = 53), the Forschungsinstitut und Natur-Museum Senckenberg, Frankfurt am Main, Germany (SFM, N = 10), the *Gothenburg* Global Biodiversity Centre at University of Göteborg, Göteborg, Sweden (GGBC, N = 40), and the Zoological Institute of the Russian Academy of Sciences, Saint-Petersburg, Russia (ZISP, N = 7) (Appendix A).

### 2.2. Gross and Microscopic Investigation

All museum specimens were preserved in 70% ethanol. Macroscopic identification of the modified scales was based on size, shape, color, and texture of the corneous epidermis. The callous scales were enlarged, swollen and lighter compared to the surrounding scales with their surface raised either entirely or in its distal or central parts. The papillary scales were easily distinguishable by color different from the surrounding areas (lighter or darker) and by visibly thicker *β*-layer comparing to the ordinary scales. For light microscopy (LM) the skin samples with the dorsal caudal, palmar and plantar scales of *A. gregori* (ZFMK 61644, male) and *L. nupta* (ZFMK 20824, male), and the precloacal scales of *L. nupta* (ZFMK 8627, subadult male?) were dehydrated in alcohol according to Romeis [26], embedded in paraffin wax at 56°, serially cut at 7–10 µm and stained with Masson’s trichrome [27].

The scanning electron microscopy (SEM) of the papillary scales was conducted using *A. gregori* (ZFMK 61644) and *L. nupta* (ZFMK 20824) as examples. We carefully removed the *β*-layer by pressing a dissecting needle against the scale surface [28]. The scales were dehydrated in absolute ethanol for approximately 5 min, incubated in a drying oven at 50 °C for two hours, sputter-coated with gold and examined using a Cambridge Stereoscan 600 scanning electron microscope, Cambridge Scientific Instruments Limited, Cambridge, UK CB1 3QH at 25 kV and a TN-2000X-Ray Analyzer, Tracor Northern, 2551 West Beltline Highway, Middleton, WI 53562, USA for EDX-analysis under magnification 50–2000×.

### 2.3. Terminology

In general taxonomy, we follow Uetz et al. [29] and Wagner et al. [30] directly for the genus *Acanthocercus*. We apply the terms “papillary scales” and “callous scales”. Following previous authors [8,25], we understand the “papillary scales” as the modified scales with papillary surface structure under loss of the outer corneous layer. This term is not equivalent to the Tölg’s “Präanale Papillarorgane” [9] we explained before and which is not equivalent to his other term, “Papillarschuppe”, which he applied to scales with femoral glands. We use the term “callous scales” understanding the latter as an equivalent of callous glands. To distinguish two basic types of epidermal holocrine specializations in lizards, we accept the terms “generation glands” and “follicular glands” [31,32]. We follow Maderson [32] in terminology of the general structure of the squamate epidermis.

## 3. Results

### 3.1. Distribution of the Modified Scales

**Figure 1:**
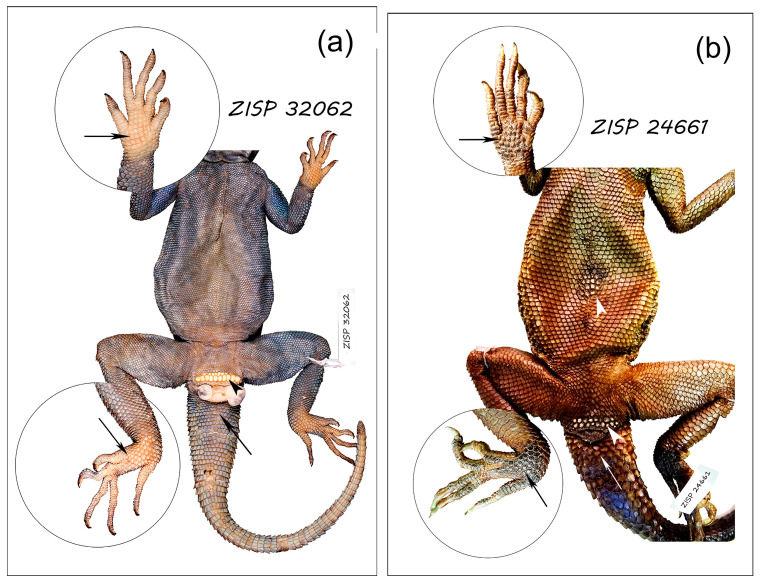
View of the ventral surface of the agamid lizards studied. (**a**) *Acantrhocercus yemensis*, ZISP 32,062 (male, mm: L = 113.9, Lcd (regenerated) = 126.7 + 5.2). Two rows of yellowish swollen callous scales are identified in front of the cloaca (arrowhead) but the mid ventral scales show no sign of callosity. The palmar and plantar scales with a lighter color, and the ventral scales of tail base have a thick corneous layer; (**b**) *Laudakia nupta*, ZISP 24,661 (male, mm: L = 160.1, Lcd (regenerated) = 221.0 + 10.7 mm). Two rows of whitish callous scales with flattened surface are identified in front of the cloaca, and a small spot of similar callous scales on the central belly is well distinguished (arrowheads); the palmar and plantar scales with a darker color and slightly uneven surface, and the ventral scales of tail base are distinguishable with thick corneous layer (arrows).

Gross observation of the integument in males of six species of *Acanthocercus* and *L. nupta* revealed the presence of the scales on the lizard’s ventrum, dorsal and ventral surfaces of the tail base, and on the palmar and plantar surfaces of the limbs that differ in shape and color from those around them (Figure 1).

A single male of *A. adramitanus* showed one row of eight clearly distinguishable callous scales in front of the cloacal vent. However, we also detected two rows of a few small weakly distinguished modified scales between the well-expressed callous row and the cloaca. The males of *A. atricollis*, *A. gregori* and *A. ugandaensis* had two or three rows in front of the vent; each consisting of two to seven swollen callous scales (Table 1), and one additional observed row. As with *A. adramitanus* only one to two rows of callous scales lying closest to the cloaca were well developed; the number of modified scales in these rows was highest compared to the other rows and the outer surface of each scale was completely worn out.

To the naked eye, in fixed animals these scales appeared as if swollen and varied from yellowish to whitish in color and from oval to polygonal in projection (Figure 1). They did not overlap each other and had more or less prismatic or rectangular shape. The “callosity” on the scales of the distant from the cloaca rows was usually visible only in their distal part or in their center. We did not detect any expressed callous modifications in other regions of the males of *Acanthocercus*, but the scales from palmar and plantar surfaces (with lighter coloration) and the dorsal and ventral scales from tail base were distinguished by the very thick corneous epidermis (Table 1; Figure 1a). After the artifactual detachment of the upper corneous layer, the surface of such scales turned out to be covered with papillae (see details below).

Although we had few females of the genus *Acanthocercus* to study (13, including 6 of *A. gregori*), in the females of *A. adramitanus* and *A. yemensis* we found callous scales in front of the vent, while in the other species callous scales were lacking (Table 1). One of the two females of *A. yemensis* had enlarged and slightly modified scales in front of the vent. The callous scales or scales with a callous-like appearance in front of the vent were clearly distinguishable in some subadult specimens of *A. atricollis*, *A. gregori*, and *A. ugandaensis.* Wealky expressed papillar scales were distinguished in the palmar, plantar and tail base regions in the females of *A. adramitanus*, *A. gregori*, *A. yemensis*, and in the only examined subadult *A. atricollis.*

The males of *L. nupta* had one to seven rows of swollen non-overlapping callous scales anterior to the cloaca, a patch of callous scales on the posterior central belly, and patches of swollen slightly overlapping and darker than surroundings the scales on the palmar and plantar surfaces. The ventral and especially the dorsal scales of the tail base were enlarged and covered with an extremely thick corneous epidermis (Figure 1b). The females of *L. nupta* lacked any modified scales in their bodies but of 10 young lizards of *L. nupta*, three specimens exhibited precloacal and abdominal callous scales as well as swollen scales on the palmar and plantar surfaces having a papillary surface after removal of the upper corneous layer.

The “callosity” was expressed unevenly within the precloacal patch of the lizards of the genus *Acanthocercus* and in the belly of *L. nupta* where the central scales were more developed than the peripheral ones. Sporadical modified scales with hypertrophied corneous epidermis surrounded the callous patches of both precloacal and abdominal regions.

No juveniles in any species of both genera showed clearly distinguished callous modifications in their integument. However, of the four juveniles of *A. gregori* three individuals were distinguished by the presence of slightly enlarged scales anterior to the cloaca.

### 3.2. Morphology of the Unspecialized Scaled Epidermis

**Figure 2:**
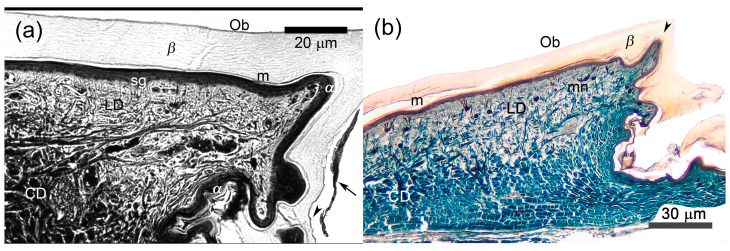
Microanatomy of unspecialized or weakly modified scales of two agamid lizards. (**a**) *Laudakia nupta*, the precloacal scale at the stage 1 of the resting phase of the sloughing cycle. Oberhäutchen is almost smooth on the outer scale surface and weakly serrated on the inner one (arrowhead). The arrow indicates the remnants of the clear layer above the new Oberhäutchen on the inner scale surface. The contact of epidermis and dermis is almost linear. (**b**) *Acanthocercus gregori*, the dorsal caudal scale at the same Stage 1. Although the contact of epidermis and dermis is almost smooth on the outer scale surface, the separate protrusions of dermis are distinguished in the top of the scale and its inner surface (arrowhead). Masson’s trichrome, parasagittal sections. Ob—Oberhäutchen; *β*—*β*-layer; m—mesos-layer; *α*—*α*-layer; LD—loose dermis, CD—compact dermis, sg—stratum germinativum; mn—melanophores.

Under LM, the structure of the unspecialized epidermis of agamid lizards resembled the one previously described for other squamates [32,34]. The sequence of epidermal layers corresponded to stage 1 of the “resting phase” of the sloughing cycle. The newly formed epidermal generation consisted, from outside inward, of the Oberhäutchen, mature *β*-layer, mesos-layer, *α*-layer and one to two layers of living cells above the stratum germinativum (Figure 2). Occasionally, remnants of the clear layer from the outer epidermal generation were visible on the surface of the new formed epidermis (Figure 2a, arrow).

The thickness, structure and staining of most of these layers varied depending upon their location within the scale. The Oberhäutchen appeared as a very thin, bright, refractive line under the microscope or a dark, thin line in microphotograph. Its surface was rather smooth on the outer side of the scales, but had weakly expressed tooth-like projections on the inner side and apparently also in the hinge regions (Figure 2a). The *β*-layer looked homogenous and chromophobic, with its maximum thickness near the top of the scale (up to 15 μm in *A. gregori* and slightly more in *L. nupta*). It became thinner on the outer posterior surface of the scale and was absent on the inner surface and in the hinge regions, where only the Oberhäutchen remained. In contrast, the eosinophilic and lamellate *α*-layer was thinnest on the outer scale surface (about 1 μm), twice as thick on the inner surface, and thickest in the hinge regions. The round-shaped germinative cells were characterized by large centrally located nuclei. Some cells in one or two living layers above the stratum germinativum showed pycnotic nuclei.

The dermis of unspecialized scales was differentiated into thinner superficial loose and thicker deep compact layers. In unspecialized dorsal scales of *A. gregori*, the ratio of loose dermis to dense dermis was 1:2, and in dorsal caudal scales it was even higher—1:5. The contact between the epidermis and dermis was relatively linear (Figure 2).

### 3.3. Morphology of the Papillary Scales

**Figure 3:**
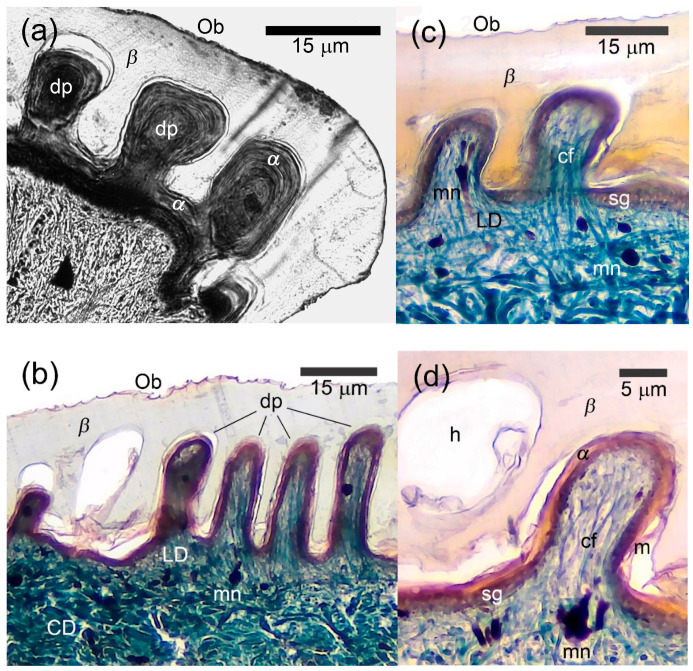
Microanatomy of papillary scales of the agamid lizards. (**a**) *Laudakia nupta*, the dermal papillae (dp) penetrated the thick *β*-layer (*β*) in semi-cross projection on the plantar scale; (**b**) *Acanthocercus gregori*, a row of straight dermal papillae (dp) in the dorsal scale; (**c**) *A. gregori*, two dermal papillae (dp) in the dorsal scale with collagen fibers extended from the loose dermis to the very tops of the papillae; (**d**) Same with Figure 3d papillae morphology under higher magnification. Top left, the hole from the papillae (h) in thick *β*-layer (*β*). See Figure 2 for other abbreviations. Masson’s trichrome, parasagittal sections except of 3a where the cut went oblique.

Under LM, the dorsal caudal, palmar and plantar scales differed in their inner microstructure from unspecialized scales by the presence of highly developed *β*-layer on their outer surface and the numerous papillae of the superficial loose dermis that deeply penetrated the epidermis (Figure 3). A parasagittal section through the dorsal caudal scales of *A. gregori* revealed 3–15 papillae per scale measuring 6–30 μm in height and 4–15 μm in basic diameter. We recorded almost identical diameter of the papillae both at the base and in their apical part (i.e., their shape was cylindrical with a diameter of 4–15 μm) (Figure 3c). It was unfeasible the measurement of the dermal papillae in *L. nupta* because of the oblique cut.

The *β*-layer was especially thick in the conjugated interpapillary areas reaching 30 μm or more in *A. gregori* and 20–30 μm in *L. nupta*. Its thickness above the papillae was twice or three times less than in the interpapillary areas (Figure 3b–d). The sequence and degree of development of the remaining layers of the epidermis (*α*-keratin and mesos layers) did not differ from those of unspecialized scales. Melanophore dendrites, blood capillaries and few fibrocytes were observed inside of the papillae. The collagen fibers extended singly or in bundles from the loose dermis to the extreme tips of the dermal papillae (Figure 3b–d). The dermal papillae varied in shape and size both in different body regions and even within a single scale, regardless of the cutting plane. They were largest on the dorsal caudal scales and smaller on the palmar and plantar ones.

**Figure 4:**
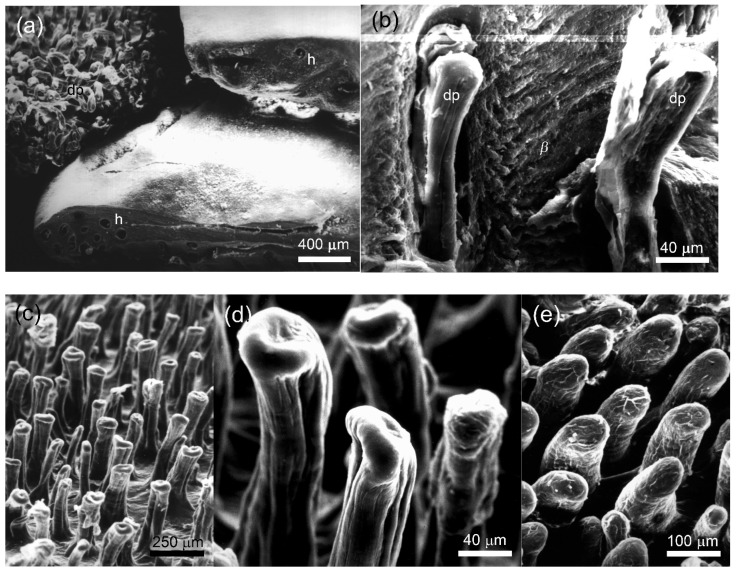
Scanning electron microscopy of the papillary scales from the dorsal surface of tail base of agamid lizards. (**a**) *Laudakia nupta*, a general view on the papillary scales. Numerous papillae (dp) are visible on the surface of the scales with the removed *β*—layer (*β*) (left). The holes in the thick *β*-layer were formed by cross-sectioning of the cavities produced by the invaginating papillae (right); (**b**) *L. nupta*, longitudinal cleavage through thick *β*-layer (*β*), showing vertical papillae (dp) covered with *α*-layer; (**c**) *L. nupta*, general view on the surface of the papillary scale without *β*-layer; (**d**) *L. nupta*, the apexes of the papillae; (**e**) *Acanthocercus gregori*, the papillae.

Under SEM, the precloacal and central abdominal scales of *L. nupta* and the precloacal scales of *A. gregori* were characterized by a general waxy appearance and by the absence of the Oberhäutchen on their surface. At the same time, the outer surface of the palmar, plantar, dorsal and ventral caudal scales was rather unevenly smooth and semitransparent. A cross section through the distal part of the scales revealed round holes, 40–80 μm in diameter (Figure 4a, two right scales) that in the sagittal or parasagittal sections appeared as long papillae that penetrated an extraordinarily thick *β*-corneous layer (Figure 4b). With the *β*-layer removed, the fields of numerous dermal papillae were distinguishable (visible) on the surface of the scales (Figure 4a, top left) which we called “papillary”.

In the dorsal scales of tail base of *L. nupta*, the papillae were oriented perpendicular to the scale surface and were relatively closely spaced (Figure 4c). They were slightly twisted, had an expanded apical tip of varying diameter (10–120 μm) and reached the maximum height of 280 μm in the dorsal caudal scales. The lateral surface of the papillae was penetrated by narrow vertical grooves (Figure 4d). The dermal papillae of *A. gregori* from the same regions were slightly sloped to the scale surface, cylindrical in shape and had smooth lateral surfaces and round apexes (Figure 4e). Their diameter varied within the same limits as in *L. nupta*, but the height did not exceed 150–180 μm. We suspect that different shapes of the papillae in the two species are the result of the quality of the fixed material and the SEM processing rather than a consequence of differences between species.

### 3.4. Morphology of the Callous Scales

**Figure 5:**
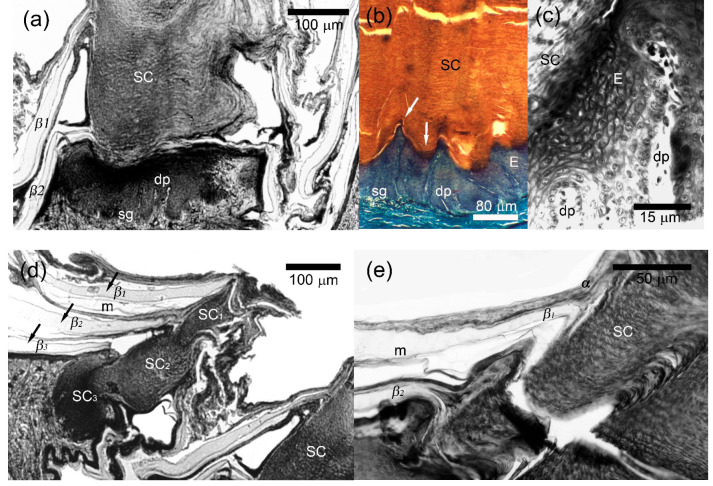
Microanatomy of precloacal callous scales of *Laudakia nupta.* (**a**) secretory corn (SC) of the central callous scale of the one of the closest to cloaca rows where almost whole scale surface contributes to corneous material release; (**b**) the basal part of the callous scale with multi-layered living epidermis (E) above the stratum germinativum (sg) and the corneous material of the secretory corn (SC) that both arranged into the popular and trough regions (arrows). The dermal papillae (dp) deeply protrude the epidermis; (**c**) two dermal papillae (dp) penetrated from the epidermis; (**d**) Secretory corn of the callous scale of the row (left) distally located from the cloaca where several packs of the corneous material (SC_1_, SC_2_, SC_3_ and molting from above) are distinguished. The corneous material of the left scale is located in its distal part while a whole scale surface contributes to the corneous material release in the right scale (the same with Figure 5a). Three mature epidermal generations (arrows) and part of the fourth molting (from above) surround the left secretory corn; (**e**) Secretory corn and neighboring α-layer of the epidermis showed a similarity in the structure and staining. See Figure 2 for other abbreviations. Masson’s trichrome. Parasagittal sections.

In the sections, the scales located anterior to the cloaca in males of *L. nupta* had the shape of truncated cones or cylinders (Figure 5a). Histological study revealed 6–20 layers of large polygonal living cells above the stratum germinativum forming epidermal papillae, with variations in thickness from 120–150 μm to 60–80 μm, respectively (Figure 5b). Living cells of polygonal shape were characterized by clear boundaries, well-distinguished nuclei and nucleoli, and basophilic cytoplasm. Some of the cells were separated by narrow but visible spaces, which were apparently artefactual results of preparation. The upper layers of the cells were distinguished by thickened membranes. The transition from the upper layers of the living cells to the basal part of the secretory corn was extremely sharp and included 2–4 layers of flattened keratinocytes with pycnotic nuclei, poorly recognized membranes and basophilic cytoplasm (Figure 5b,c).

The secretory corneous material consisted of lamellate secretion that stained eosinophilic with Masson’s trichrome. It was characterized not only by horizontal lamellar packing but also by vertical columns of barely discernible cells (Figure 5b). At higher magnification, the arrangement of these cells somewhat resembled the stacking of tetradecahedral cells in the stratum corneum of mammals (Figure 5c, top left). The corneous material of the secretory corn mirrored the arrangement of ridges and sulci of the living layers (Figure 5b). It partially retained the cellular structure and contained the remnants of nuclei (Figure 5a,d). Outside, the secretory corns were surrounded by several generations of epidermis with a normal sequence and morphology of layers (Figure 5a,d,e). Several generations of the secretory product could also be seen in callous scales of more distant rows from the cloaca (where callosity appeared only at the distal end of the scale: Figure 5d).

An irregular contact surface was seen between epidermis and dermis since numerous dermal papillae up to 20–120 μm in length, extended deep into the epidermis and most of them penetrated the epidermis in the ridge regions (Figure 5b).

In their histological appearance, the abdominal callous scales resembled the precloacal ones. However, layers of large polygonal cells above the stratum germinativum were reduced in thickness, the callous corn was visibly shorter, and the dermal papillae were less numerous and prominent compared to the precloacal ones.

## 4. Discussion

### 4.1. Papillary Scales and Their Functional Value

Papillary scales of the agamid lizards were previously mentioned only from gross observations [5,7,8]. Using LM and SEM, we first described their structure, which is characterized by presence of a thick and resistant corneous layer in the epidermis and highly developed papillae in the dermis.

The enhanced development of the dermal papillae is known in strongly cornified areas subjected to friction and in glabrous areas of the integument of some mammals (sperm whales, dolphins, pigs, elephants), and humans [35,36,37,38]. They are also described for the callous scales in the ventral integument of Asian ring-tailed agamas and some other agamid lizards [1,13,15]. A thick layer of corneous material is produced in these cases during different processes: *β*-cornification in the papillary scales and α-keratinization in mammals and in the callous scales of lizards. Although the *α*-keratins and corneous *β*-proteins are encoded at different chromosome loci and belong to different proteins families [39,40], the participation of dermal papillae in overgrowth of the *β*-corneous and *α*-keratin layers seems similar. It includes an increase in the internal production surface of proliferating germinative cells, the formation of a pool of proliferating and differentiating cells in cycle at the base of dermal papillae, as well as enhanced trophism of these processes due to the presence of blood capillaries [36].

Earlier researchers provided additional functional interpretations for the strongly developed dermal papillae in the scales of agamid lizards. Iohanssen [5] suggested that papillary formations contribute to strengthening the connection between the *β*-layer and the underlying epidermal layers. Keeping in mind that there is a mechanical “weak zone” in the mesos-layer, where a transition between *β*-corneous proteins to *α*-keratins takes place [41,42], it can be assumed the dermal papillae can act as “anchors”, creating a kind of zip-effect and preventing a detachment of a thick *β*-layer. The trend of greater development of papillary scales toward the distal parts of the limbs and tail observed in species of the “*caucasia*” and “*stellio*” complexes may be associated with the mechanical stress experienced by these body areas during a petrophilic lifestyle of lizards [23,43,44,45].

Because the thick corneous layer may shield external stimuli, we suspect that specific receptors may be found in the dermal papillae of the agamis scales. For example, the mechanoreceptors (Herbst and Grandry corpuscles) located in cylindrical dermal papillae passing through the massive *β*-corneous layers were described in the bill tip of *Anas platyrhynchos* [46,47] and numerous Merkel’s corpuscles were recorded in the dermal papillae penetrating the thick permanently erasable α-layer of the epidermis in humans and primates [48].

Suggestions about the role of dermal papillae in increasing the mechanical strength of the skin and participation in mechanoreception are consistent with data on the skin of cetaceans. The dermal papillae in the skin of deep-diving sperm whales provide a tighter adhesion between the dermis and epidermis [36]. In dolphin skin, strongly pronounced papillary ridges may provide support for the skin, which experiences stress from hydrodynamic forces, and may help transmit mechanical stimuli [49,50].

So far among Squamata, the presence of highly developed dermal papillae in large and hard scales remains a unique feature of Asian ring-tailed agamas and the species of *Acanthocercus.* Large and hard scales are present in the tail of all species of the genus *Uromastyx* (commonly known as spiny-tailed lizards) and Madagascar iguanas of the genus *Oplurus*, but papillary scales have seemingly not yet been found in either of them. It is possible that hypertrophy of the *β*-layer in these lizards was achieved in various ways. The scalation of Squamata shows other examples of achieving the same morphological pattern by different routes. The highly overlapping scales of the snakes are formed with the condensation of dermal cells in the apical part of the scale as a possible growth point [51,52] while in skinks they are shaped by apical growth of the scale tip composed of a solid rod of *β*-cells [53]. These facts are remarkable and confirms the well-known principle: “Nature is prodigal in variety, but niggard in innovation”, perfectly expressed by Milne Edwards in the middle of the 19th century [54].

### 4.2. Callous Scales: Affinity to Epidermal Glands, Functional and Taxonomic Value

Histological study of the callous scales of *L. nupta* confirmed that the secretory corneous material is formed from the hyperplasia of differentiating corneous layers of the epidermal generation [9,10,14,15]. Moody [17] considered the precloacal/abdominal callous glands of agamid lizards as only superficially similar to the “generation-type” gland which we also support. The formation of the secretory material in true generation glands occurs in association with periodic skin-shedding [19,55,56]. When molting is delayed, several generation of the secretory material can be accumulated. This phenomenon has been marked in the “protruding glands” of the cordylid lizards, where more than 10 “stacked” generations of glandular material were produced during once-a-year “pan-body shedding” [57,58,59]. In the callous integument of *L. nupta*, the secretory corneous material had a relatively monolithic structure in the central, most developed callous scales while it accumulated in several packs in the scales of the distal position (comp. Figure 5a,d). On our opinion, the callous scales seem to represent some intermediate state between the “simplest” generation glands and true follicular glands, where the processes of production and release of glandular material occur independently. van Wyk and Maderson [57] have classified such characteristic structures as a variation of the generation glands, where the production of secretion is *temporally asynchronous* with the “pan-body shedding cycle”. A possible “intermediate” status of callous scales is also supported by the strong development of the basal portions of dermal papillae (Figure 5a,c). The perforation by dermal papillae of the overlying germinal and living daughter layers of the epidermis in *L. nupta* to some extent resembles the beginning of the process of compartmentalization or “alveolization” of the body of follicular glands, which was shown in some iguanids and geckos [60,61].

**Figure 6:**
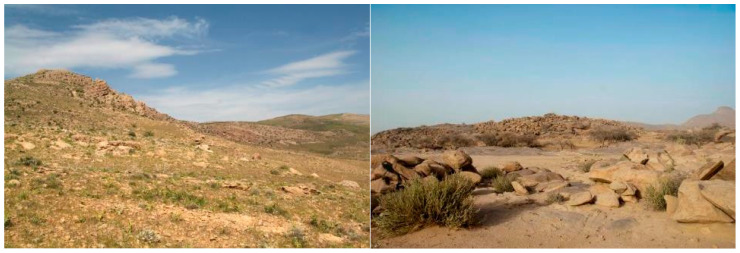
(**a**) habitat of *Laudakia nupta*, Persepolis, Fars province, Iran, April 2006. Photo by R. Nazarov; (**b**) habitat of *Acanthocercus adramitanus*, Dalagan, vicinity of Abha, Saudi Arabia, April 2015.

Enhanced keratinization of certain areas of lizard body can be explained by the mechanical stress exerted on these body regions and the effect of surface layer abrasion, which stimulates proliferation and cornification in the epidermis [23,62]. All authors are inclined to believe that the secretion of callous scales plays a role in territorial behavior and the formation of hierarchical relationships in agamid lizards inhabiting hard substrates [13,15,23]. Indeed, *L. nupta*, as well as *A. adramitanus* and *A. yemensis* inhabit the rocky habitats (Table 1, Figure 6). In these two species of *Acanthocercus*, the callous scales occur even in some females (Table 1) [63]. Since callous scales are more male characteristic [14,15,23], the presence of callous-like scales in front of the vent and/or weakly recognizable papillar scales in some subadult specimens (Table 1) may indicate belonging to males.

On the other hand, it appears noteworthy that except for *A. minutus*, which is a ground-living inhabitant of dry savannah and woodland, the other species (*A. atricollis*, *A. gregori*, and *A. ugandaensis*) are tree dwelling. Nevertheless, their males also have well-defined callous scales in front of the cloaca (Table 1). In this case, the secretion process probably relies on different mechanisms. They were described as “presumptive generation glands” in gerrhosaurid lizards without an expressed mature generation of secretory mass [59]. An evidence of desiccation of the secretion of generation glands without mechanical wearing was shown for *Cordylus macropholis* [64]. It is not known, whether this process developed independently or in parallel with the abrasive one.

The possibility of using the epidermal glands in lizard taxonomy and phylogeny is still vague. Callous glands were described in the lizards of the families Agamidae [9,10,14,15,16,17,65] and Cordylidae [20,57,58,59,66]. The “simplest” generation glands (*β-* or escutcheon) were found among geckos in Diplodactylidae, Eublepharidae, Gekkonidae, and Sphaerodactylidae [31,67,68,69,70,71], as well as in Gerrhosauridae [59] (as “presumptive generation glands”) and Opluridae [24]. The follicular glands have long been regarded as an important feature of gekkotan lizards [61,72,73], agamid lizards (see review [74], including Uromastycinae [75,76], Cordylidae [66], Crotaphytidae [60,77], and Lacertidae [22,78,79]. They also were recorded in Amphisbaenidae [80] and Gerrhosauridae [81]. The epidermal *α*-glands described recently in tropidurine lizards [82], actually more closely resemble the escutcheon generation glands, in particular, oplurines which have modified innermost layers of the epidermal generation [24] and seemingly do not represent a special type.

Analysis of the studies cited above, shows that different types of epidermal glands were found in species of different phylogenetic lineages. Several types of the glands can occur in the same lineage, and even in the same species. Such facts support the view of independent development of epidermal glands in different phyletic groups [83]. Seemingly, it is a consequence of the intrinsic ability of the epidermis in terrestrial vertebrates to secrete keratin proteins and extracellular lipids into the environment through various pathways [39,84]. Taking terrestrial vertebrate keratinization as the primary uncontrolled semiochemical source and discussing the evolution of dermal glands, Maderson [56] proposed that the abraded regions of the epidermis were protoadaptations for the discreet “glands”. Such a circumstance suggests being cautious when considering the epidermal glands as a reliable approach for solving taxonomy problems.

Returning to the analogy with the skin of mammals, we note that their callous structures are formed by the *α*-pattern of soft cornification [40], while the origin of the secretory product in the callous scales of agamids remains unclear. A study the callous glands in the species of the genus *Paralaudakia* pointed to a certain morphological and tinctorial similarity of emerging secretory product to the process of *α*-keratinization and a histochemical resemblance to the mesos-layer [14]. In terms of lamellar appearance and affinity for acidic dyes, mature callous gland material in some way resembles the *α*-layer on the outer side of the papillary scales and the *α*-layer of the scale areas surrounding the secretory plug (comp. Figure 3a and Figure 5e). This similarity with the preservation of the cell contours and the remnants of nuclei in the secretory corn bears certain resemblance to the histological picture of parakeratosis in humans and mammals [85]. Regardless of future results, it is possible to treat the existing similarity in the *gross* pattern of callous patches in the integument of lizards and mammals as an example of convergent development.

## 5. Conclusions

Two types of modified scales have been described in the integument the agamid lizards—the callous and papillary scales. Both types of scales are localized mainly on the ventral side of the body (papillary ones are also on the dorsal surface of the base of the tail) and show a marked sexual dimorphism, being presented and more developed in the males and weakly developed or even absent in females. Morphologically, the structural pattern of callous and papillary scales is the same. They distinguish by high development of the corneous layer (containing *α* -keratin in the former and *β*-proteins in the latter) and expression of the dermal papillae, which promotes the hyperplasia of the corneous layer. Peculiarities of scale topography and the rate of their development in the studied species confirm a suggestion on their functional connection with territorial lifestyle and hard substrate preference of the lizards.

Our study raised more questions than we could answer. How do dermal papillae develop in ontogeny? Since well-developed papillary scales were absent in the integument of juvenile lizards, it is likely that these scales grow extremely fast. Is there some “relationship” between papillary and callous scales, since their general structural pattern is similar? How objective are the functional analogies in the interpretation of the morphological similarity of the modified scales in lizards with similar formations in the skin of other vertebrates outlined by us? We hope that future research including TEM and immunohistochemical analysis could help to resolve many of these issues.

## Figures and Tables

**Table 1:** Distribution of the modified scales (callous and papillary) in the integument of six species of the genera *Acanthocercus* and *Laudakia nupta:* N—number of the specimens examined, M—males, F—females, subad M?—probably young males, subad F?—probably young females, juv—juveniles before the first wintering; PC—precloacal scales, MV—midventral scales, P/P—palmar and plantar scales, C—caudals (dorsal and ventral), (+) present, (–) absent, ˂—weak development, ?—difficult for sex determination. Data on type of habitats are borrowed from [33].

Species Number of Specimens Studied in Parentheses	Sex/Age	N	Callous Scales			Papillary Scales		Habitats
			PC		MV	P/P	C	
			Rows	Scale/Row	+/–	+/–	+/–	
*Acanthocercus adramitanus* (2)	M	1	1	8	–	+	+	Rocky areas
	F	1 *	1	10	–	˂	˂	
*A. atricollis* (7)	M	5	2–3	3–6	–	+	+	Savanna, woodland, forest clearings
	F	1	–	–	–	–	–	
	subad M?	1	1	?	–	˂	˂	
*A. gregori* (26)	M	13	2–3	2–6	–	+	+	Grassland, savanna
	F	6	–	–	–	˂	˂	
	subad M?	4	?	?	–	–	–	
	subad F?	2	–	–	–	–	–	
	juv	1	? **	–	–	–	–	
*A. minutus* (7)	M	2	3–4	1–6	–	+	+	Savanna
	F	2	–	–	–	–	–	
	juv	2	? **	–	–	–	–	
	juv	1	–	–	–	–	–	
*A. ugandaensis* (7)	M	3	2–3	4–7	–	+	+	Forest, artificial
	F	1	–	–	–	–	–	
	subad M?	2	2	4–6	–	–	–	
	subad F?	1	–	–	–	–	–	
*A. yemensis* (3)	M	1	2	8, 10	–	+	+	Rocky areas
	F **	2	2	8	–	˂	˂	
*Laudakia nupta* (58)	M	33	1–7	8–10	+	+	+	Rocky areas, shrubland
	F	12	–	–	–	–	–	
	subad M?	3	+	+	+	+	+	
	subad F?	7	–	–	–	–	–	
	juv	3	–	–	–	–	–	

Comments: * additionally, this female had two rows with enlarged, but poorly developed callous-like scales; ** the scales were enlarged but without clear callous appearance.

## Data Availability

Data are contained within the article.

## Appendix A

List of the specimens examined:

*Acanthocercus adramitanus* (2): ZISP 32061, 32221.
*A. atricollis* (7): ZFMK 2691, 2692, 7423, 7424, 18394, 29397; ZISP 19699.
*A. gregori* (26): ZFMK 2685, 2689, 2690, 15865–15868, 19161, 20854, 21073–21076, 21078, 21079, 21081–21086, 41655, 61661, 61662, 61644, 61665.
*A. minutus* (7): ZFMK 2683, 2684, 2686–2688, 16229, 16230.
*A. ugandaensis* (7): ZFMK 55710–55716.
*A. yemensis* (3): ZISP 32062, 32115, 32220.
*Laudakia nupta* (58): ZFMK 2682, 7924, 8627, 8628, 20824–20826; GNM Museum numbers: Re. ex. 5521–5560; SMF 63217–63220, 63222, 63224, 63225, 63228–63230; ZISP 24661.
